# Altered T Lymphocytes Mitochondrial Function and Inflammatory Factors of Peripheral Blood in HIV Patients With Mycobacterial Infection

**DOI:** 10.1111/jcmm.70832

**Published:** 2025-09-11

**Authors:** Mengyan Wang, Xiaotian Dong, Hu Wan, Jinchuan Shi, Lu Hui, Wei Chen, Shourong Liu, Jun Yan

**Affiliations:** ^1^ Department II of Infectious Diseases, Xixi Hospital of Hangzhou Hangzhou Xixi Hospital Affiliated to Zhejiang University of Traditional Chinese Medicine Hangzhou China; ^2^ Department of Laboratory Medicine, the First Affiliated Hospital, College of Medicine Zhejiang University Hangzhou China

**Keywords:** human immunodeficiency virus, inflammatory factors, low mitochondrial membrane potential, mitochondrial mass, tuberculosis

## Abstract

To characterise T‐cell immunity and inflammatory profiles in HIV patients with mycobacterial co‐infections. This study enrolled 41 HIV patients co‐infected with *Mycobacterium tuberculosis* (HIV‐TB, *n* = 27) or non‐tuberculous mycobacteria (HIV‐NTM, *n* = 14), along with 30 controls (20 HIV‐monoinfected, 10 post‐treatment) from a single centre. Flow cytometry quantified T‐cell subsets (CD3 + CD4+, CD3 + CD8+, CD28+ subsets), mitochondrial parameters (mass [MM], low membrane potential [MMP‐low%]) and cytokines (IFN‐γ, IL‐2/4/6/10/17A, TNF‐α). Co‐infected groups showed reduced T‐cell counts versus HIV‐monoinfected controls (*p* < 0.05). Elevated MMP‐low% in CD3 + CD4+/CD28+ T cells indicated mitochondrial dysfunction in co‐infected patients (*p* < 0.05). HIV‐TB patients exhibited higher CD3 + CD4+/CD28+/CD8+ T‐cell MM than HIV‐NTM (*p* < 0.05), while HIV‐NTM demonstrated greater MMP‐low% (*p* < 0.05). Proinflammatory cytokines (IFN‐γ, IL‐6, IL‐17A) inversely correlated with CD4+ counts and MM, but positively with CD8 + CD28+ MMP‐low%. MMP‐low% in CD3 + CD4 + CD28+ T cells and IL‐2 differentiated IRIS/non‐IRIS cases (*p* < 0.05), with combined AUC = 0.834 for IRIS prediction (*p* = 0.001). HIV/mycobacterial co‐infection exacerbates T‐cell depletion and mitochondrial dysfunction, with HIV‐NTM showing more severe impairment. MMP‐low% and IL‐2 may serve as biomarkers for IRIS risk stratification.

## 1 Introduction

According to the WHO global data, in 2024, there were 10.8 million new tuberculosis cases in the world, with an incidence rate of 134 per 100,000. This includes 6.1% of HIV‐infected patients, of which 1.25 million have died, including 161,000 patients with HIV infection [1]. The AIDS phase of HIV‐infected patients is a high‐risk period for active tuberculosis [2]. In addition, tuberculosis is one of the most common infectious diseases in the world [3]. Synergies of HIV and *Mycobacterium tuberculosis* (Mtb) co‐infection amplify the disease burden. This synergy is centred on immunological deterioration [4]. In recent years, the incidence of nontuberculous mycobacteria (NTM) has been increasing year by year, especially in immunocompromised HIV‐infected patients (PLWHA) [5].

Immune reconstitution inflammatory syndrome (IRIS) is an exaggerated inflammatory response to an existing pathogen or its antigen, meaning the recovery of the body's immune system associated with the initiation of antiretroviral therapy (ART) in patients with AIDS due to a human immunodeficiency virus (HIV) infection. *M. tuberculosis* is the most common among the mycobacteria, and non‐tuberculous mycobacteria have been becoming more and more common recently [6]. The purpose of this study was to investigate the immune and inflammatory characteristics of HIV patients with mycobacterial infections.

## 2 Materials and Method

### 2.1 Subjects

During February 2022 and July 2024, 27 cases of tuberculosis and 14 cases of non‐tuberculous mycobacteria were confirmed in Hangzhou Xixi Hospital, and the control group (10 patients were followed up after treatment, and 20 patients were infected with HIV only) was enrolled in this study. Inclusion criteria: Patients over 16 years of age with a diagnosis of HIV combined with active mycobacterial infection. The diagnosis of HIV infection is based on relevant guidelines: The diagnosis of HIV infection meets one of the following conditions: (1) positive HIV antibody screening test, positive HIV supplemental test (positive antibody supplemental test or positive nucleic acid qualitative test or nucleic acid quantification greater than 5000 copies/mL); (2) Have an epidemiological history or AIDS‐related clinical manifestations and have two positive HIV nucleic acid tests; (3) Be born to an HIV‐infected mother and a positive HIV isolation test. Diagnosis of mycobacterial infection is based on laboratory data (microscopy on sputum acid‐fast stained smears and/or mycobacteria in sputum/blood/tissue mycobacterial culture and/or mycobacteria in GeneXpert and/or NGS for bronchial lavage). Exclusion Criteria: Other immune diseases. Data on age, gender, HIV‐RNA, ART regimen, anti‐NTM/TB regimen, duration of fever before and after treatment, IRIS (immune reconstitution inflammatory syndrome), prognosis and corticosteroid or immunosuppressant therapy were collected. The timing of lymphocyte and cytokine testing was conducted at the initial infection with TB/NTM, and 10 patients in the follow‐up group were tested after the post‐treatment of anti‐infective treatment. All comparisons between pre‐ and post‐treatment exclude deceased patients and are limited to individuals who received treatment for more than 3 months.

### 2.2 T Lymphocyte Count and Mitochondrial Indicator Measurements by Flow Cytometry

Flow cytometry was used to detect the level of peripheral blood lymphocytes and mitochondrial membrane potential. The mitochondrial membrane potential (MMP) of peripheral blood T lymphocytes was detected by NovoCyte flow cytometry. Data was analysed, and the detection reagents were CD3 detection reagent (FITC, UCHT1), CD45 detection reagent (PerCP‐Cy5.5, HI30), CD4 detection reagent (PE‐Cyanine7, SK3), CD28 detection reagent (PE, CD28.2) CD8 detection reagent (APC‐Cy7, SK1) [Ubiquitous Peptide Biotechnology (Zhejiang) Co. Ltd.], detection index: CD3+ T lymphocytes (T) and its subsets CD3 + CD4+ T lymphocytes, that is helper T lymphocytes (Th), CD3 + CD8+ T lymphocytes, that is killer T lymphocytes (Tc) and CD3 + CD8 + CD28+ T lymphocytes, CD3 + CD4 + CD28+ T lymphocytes, absolute value, mitochondrial mass, mitochondrial membrane potential. These dyes specifically bind to green fluorescent dyes that bind to the mitochondria of living cells (can be excited at 633 nm). Graphical analysis is presented in the Appendix S1. Among them, mitochondrial mass (MM) indicates the content of effective protein in the respiratory chain of the inner mitochondrial membrane, which represents the actual upper limit of the metabolic capacity of immune cells and reflects the actual strength of the body's immunity. The percentage of cells with low mitochondrial membrane potential (MMP‐low%) refers to the percentage of cells with low MMP in the total number of such cells, representing the actual state of immune cell metabolism.

### 2.3 Cytokine Assays by Flow Cytometry

Seven cytokines were quantitatively detected in serum by double‐antibody sandwich fluorescence luminescence and liquid suspension microarray technology, including interleukin‐2 (IL‐2), interleukin‐4 (IL‐4), interleukin‐6 (IL‐6), interleukin‐10 (IL‐10), interleukin‐17A (IL‐17A), tumour necrosis factor α (TNF‐α) and interferon‐γ (IFN‐γ). Monoclonal antibodies to seven cytokines, including IL‐2, IL‐4, IL‐6, IL‐10, IL‐17A, TNF‐α and IFN‐γ, were conjugated with different fluorescence‐encoded magnetically encoded microspheres to prepare multi‐cytokine magnetically encoded microspheres with different encodings. A certain amount of microsphere dilution was mixed with the sample to be tested. After the first incubation stage, the cytokines in the sample to be tested were specifically combined with the antibody fixed by the magnetically encoded microsphere to form an antigen‐antibody‐magnetically encoded microsphere complex. In the second stage, fluorescent protein‐labelled antibodies were added and incubated to form antibody‐antigen‐fluorescent protein‐labelled antibody complexes. The number of fluorescent antibodies on different magnetically encoded microspheres could reflect the concentration of each cytokine in the samples to be tested. Finally, the fluorescent antibody signal was captured and decoded by a fractional luminescence analyser, and the content of each cytokine in the sample (pg/mL) was quantified according to the intensity of the fluorescence signal and the calibration curve of each index.

Patients were divided into IRIS and non‐IRIS subgroups according to whether they had IRIS during diagnosis and treatment. According to the International Network for the Study of HIV‐associated IRIS [7], case definition for TB‐associated IRIS in resource‐limited settings: Antecedents: TB‐diagnosis according to WHO guidelines before starting ART; TB should have stabilised or improved before starting ART. Clinical criteria: New enlarging lymph nodes, cold abscesses or other focal tissue involvement; new or worsening radiological features of TB; new or worsening CNS tuberculosis; new or worsening serositis. Exclusion of alternative causes: Failure of TB treatment (non‐compliance or resistance); other opportunistic infection or neoplasm; drug toxicity reaction. Forty‐one patients co‐infected with HIV and mycobacteria were excluded from treatment failure, a total of 40 cases divided into 11 cases in the IRIS group and 19 cases in the non‐IRIS group.

### 2.4 Statistical Analysis

Discrete data are expressed in counts (percentages), and continuous data are expressed as normally distributed ± standard deviations, medians of non‐normal distributions and interquartile ranges. Compare the differences between continuous data using the Mann‐Whitney *U*‐test (non‐parametric) or the Student t‐test (normally distributed). Data before and after treatment were tested using paired samples for non‐parametric tests. A chi‐square test with continuity correction was used to assess differences between categorical data. The area under the curve (AUC) of the receiver operating characteristic (ROC) curve was calculated to evaluate the performance in the prediction of disease. All statistical analyses were performed using SPSS, and *p* < 0.05 was considered statistically significant.

## 3 Results

### 3.1 Demographic Characteristics

As shown in Table 1, a total of 27 patients with confirmed tuberculosis were included, including 26 males and one female, with an average age of 42.85 ± 9.453 years, 9/27 patients with HIV‐RNA below the detection line, and 21/27 patients who had received antiviral therapy. Three out of 27 patients died. There were 14 cases of nontuberculous mycobacteria, 12 males and two females, with an average age of 39.00 ± 14.11 years; 14/14 patients had received antiviral therapy, and 9/14 patients had HIV‐RNA lower than the detection line. Two out of 14 patients died. There were 20 HIV‐infected patients in the control group, including 20 males, with an average age of 39.20 ± 11.23 years; 20/20 patients had received antiviral therapy, and 20/20 patients had HIV‐RNA levels lower than the lower line of detection. Nontuberculous mycobacterium was mainly infected with *Mycobacterium avium* ‐intracellular complex. All confirmed patients received antimycobacterial therapy, and treatment options varied due to individual differences.

**TABLE 1:** Clinical characteristics of patients.

Characteristics	HIV‐TB patients (*N* = 27)	HIV‐NTM patients (*N* = 14)	HIV patients (*N* = 20)
Age, years	42.85 ± 9.453	39.00 ± 14.11	39.20 ± 11.23
Gender ratio (M:F)	26:1	12:2	20:0
Species, *n* (%)			
*Mycobacterium tuberculosis*	27 (100%)	—	—
*Mycobacterium avium* ‐intracellular complex, MAC	—	11 (78.57%)	—
*Mycobacterium genavense*	—	1 (7.14%)	—
*Mycobacterium gordonae*	—	1 (7.14%)	—
Mycobacterium thermoresistible	—	1 (7.14%)	—
HIV‐RNA, ≤ 100 copies/mL	9 (33.33%)	9 (64.28%)	20 (100%)
Treatment, *n* (%)			
ART	21 (77.77%)	14 (100%)	20 (100%)
H + E + Rb	3 (11.11%)	—	—
H + Z + E + Rb	2 (7.4%)	—	—
H + Z + E + *R*	2 (7.4%)	—	—
H + Z + E + L	4 (14.81%)	—	—
H + Z + E + M	4 (14.81%)	—	—
H+ Rb + M + LZD	2 (7.4%)	—	—
H + Z + E + Rb + M	7 (25.92%)	—	—
H + Z + E + *R* + M	1 (3.70%)	—	—
H + Z + E + Rb + M + LZD	2 (7.4%)	—	—
E + M + AZM	—	1 (7.14%)	—
E + Rb + M + AZM	—	3 (21.42%)	—
E + Rb + M + A	—	3 (21.42%)	—
E + M + A + AZM	—	2 (14.28%)	—
E + M + A + AZM + LZD	—	2 (14.28%)	—
H + E + *R* + M + A	—	3 (21.42%)	—
Number of deaths	3 (11.11%)	2 (14.28%)	—

Abbreviations: A, amikacin; AZM, azithromycin; E, ethambutol; H, isoniazid; L, levofloxacin; LZD, linezolid; M, moxifloxacin; R, rifampicin; Rb, rifabutin; Z, pyrazinamide.

### 3.2 Alterations in Peripheral Blood T Lymphocytes Function and Inflammatory Factors in Different Groups

**FIGURE 1:**
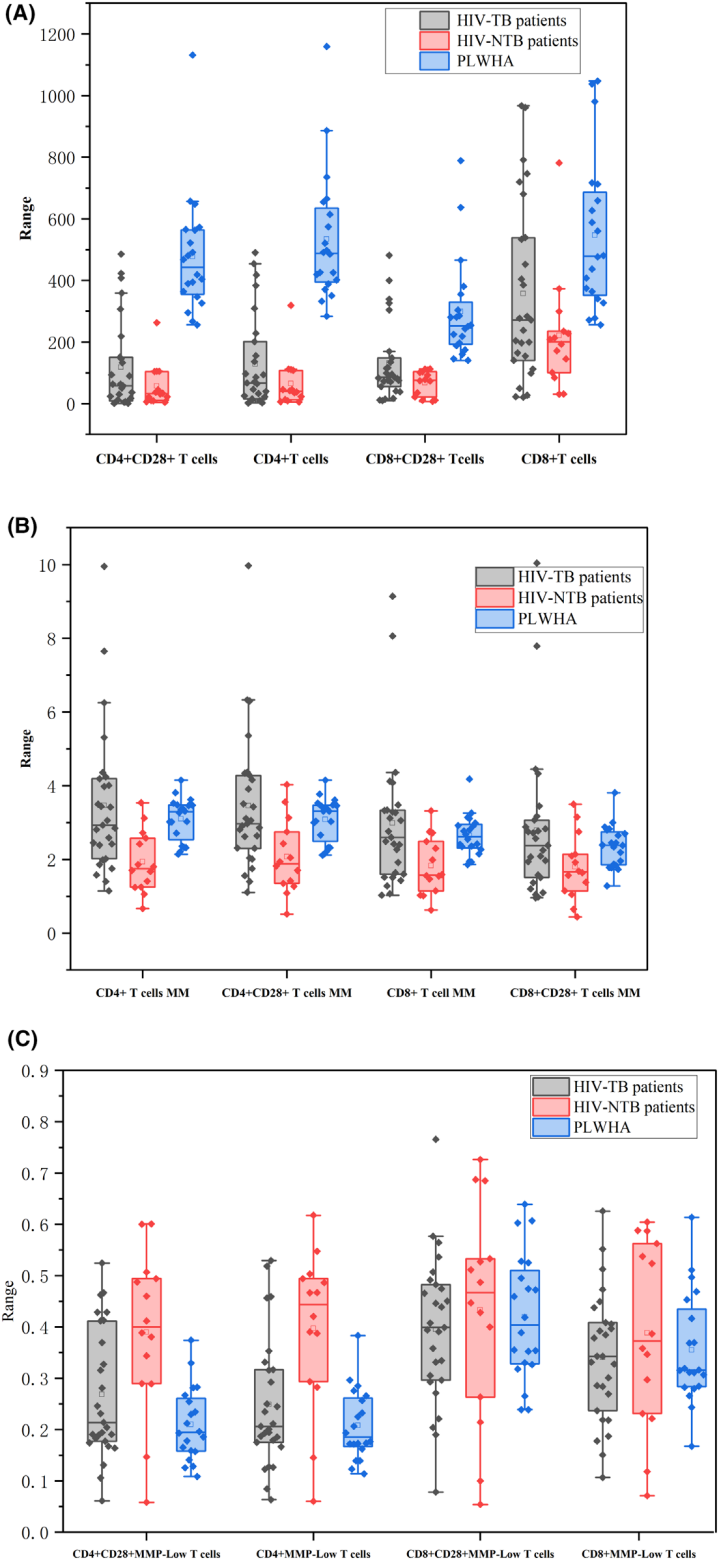
Significantly different distributions of peripheral blood T lymphocytes in different groups. Different lymphocyte count (A), altered MM (B) and different MMP‐low% (C) of PLWHA, HIV‐NTM patients and HIV‐TB patients. PLWHA, immunocompromised HIV‐infected patients; HIV, human immunodeficiency virus; MM, mitochondrial mass; MMP‐low%, percentage of low mitochondrial membrane potential.

The CD3 + CD4 + T cell count, CD3 + CD4+ T cell MM, CD3 + CD4 + CD28+ T cell count, CD3 + CD4 + CD28+ T cell MM, CD3 + CD8 + T cell count, CD3 + CD8+ T cell MM, CD3 + CD8 + CD28+ T cell count in HIV‐TB patients and HIV‐NTM patients were significantly lower than that in HIV‐infected patients (*p* < 0.05), as shown in Table 1. The MMP‐Low% of CD3 + CD4+ T cell and MMP‐Low% of CD3 + CD4 + CD28+ T cell in HIV‐TB patients and HIV‐NTM patients were significantly higher than those in HIV‐infected patients (*p* < 0.05), as shown in Table 1. In addition, the MM of CD3 + CD4+ T cell, MM of CD3 + CD4 + CD28+ T cell, and MM of CD3 + CD8+ T cell in HIV‐TB patients were significantly higher than those HIV‐NTM patients (*p* < 0.05). The MMP‐Low% of CD3 + CD4+ T cells and MMP‐Low% of CD3 + CD4 + CD28+ T cells were significantly lower than those in HIV‐NTM patients (*p* < 0.05), as shown in Table 1 and Figure 1A–C. The levels of IFN‐γ, Interleukin 17A, Interleukin 2, Interleukin 4, Interleukin 6 and TNFα in HIV‐TB patients and HIV‐NTM patients were significantly higher than those in HIV‐infected patients (*p* < 0.05), as shown in Table 2.

**TABLE 2:** Comparison of peripheral blood T lymphocyte mitochondrial function and inflammatory factors between HIV patients with mycobacterial infection and HIV‐infected patients.

Groups	HIV‐TB patients (*N* = 27)	HIV‐NTM patients (*N* = 14)	HIV patients (*N* = 20)	*p*
CD3 + CD4 + T cell count (pcs/μL)	66.70 (15.19, 201.20)^a^	39.95 (11.87, 108.69)^a^	488.11 (391.75, 644.51)	0.001
MM of CD3 + CD4+ T cell	2.93 (2.02, 4.19)^a^	1.75 (1.25, 2.62)^a^, ^b^	3.29 (2.45, 3.48)	0.002
MMP‐Low% of CD3 + CD4+ T cell	20.61% (17.50%, 31.69%)^a^	44.36% (29.07%, 49.65%)^a^, ^b^	18.49% (16.46%, 26.33%)	0.002
CD3 + CD4 + CD28+ T cell count (pcs/μL)	57.86 (8.57, 150.87)^a^	32.40 (9.71, 104.17)^a^	442.94 (350.82, 564.57)	0.001
MM of CD3 + CD4 + CD28+ T cell	2.97 (2.30, 4.28)^a^	1.88 (1.33, 2.84)^a^, ^b^	3.31 (2.41, 3.48)	0.008
MMP‐Low% of CD3 + CD4 + CD28 + MMP‐Low T cell	21.39% (17.71%, 41.18%)^a^	40.02% (28.95%, 49.74%)^a^, ^b^	19.45% (15.76%, 26.38%)	0.003
CD3 + CD8 + T cell count (pcs/μL)	271.75 (140.51, 539.16)^a^	200.57 (96.73, 251.28)^a^	478.57 (346.15, 699.18)	0.001
MM of CD3 + CD8+ T cell	2.60 (1.60, 3.34)^a^	1.58 (1.12, 2.55)^a^, ^b^	2.62 (2.29, 2.97)	0.016
MMP‐Low% of CD3 + CD8+ T cell	34.27% (23.69%, 40.87%)	37.24% (22.87%, 56.86%)	31.57% (28.29%, 44.38%)	0.629
CD3 + CD8 + CD28+ T cell count (pcs/μL)	85.36 (55.14, 148.50)^a^	76.05 (19.11, 105.06)^a^	252.77 (190.42, 342.33)	0.001
MM of CD3 + CD8 + CD28+ T cell	2.38 (1.51, 3.07)	1.66 (1.13, 2.29)	2.39 (1.85, 2.78)	0.104
MMP‐Low% of CD3 + CD8 + CD28+ T cell	39.92% (29.63%, 48.26%)	46.70% (25.10%, 57.10%)	40.37% (32.75%, 51.76%)	0.626
IFN‐γ	1.48 (0.83, 3.38)^**a**^	1.39 (1.06, 3.42)^a^	0.64 (0.52, 0.76)	0.001
Interleukin10	3.72 (2.44, 8.61)	3.31 (2.62, 18.76)	2.04 (1.36, 4.64)	0.109
Interleukin17A	1.21 (0.96, 3.01)	1.49 (1.07, 1.58)^a^	1.11 (0.99, 1.27)	0.019
Interleukin2	0.55 (0.33, 0.79)^a^	0.55 (0.36, 1.60)^a^	0.23 (0.18, 0.26)	0.001
Interleukin4	1.06 (0.83, 1.52)^a^	0.95 (0.77, 1.140)	0.76 (0.63, 0.86)	0.014
Interleukin6	10.43 (5.81, 36.51)^a^	11.74 (5.87, 28.36)^a^	4.01 (3.64, 5.53)	0.002
TNFα	2.94 (2.14, 4.92)^a^	2.36 (1.95, 7.23)	0.81 (0.64, 1.26)	0.001

Abbreviations: NTM, nontuberculous mycobacteria; TB, tuberculosis.

^a^ Compared with HIV patients *p* < 0.05.

^b^ Compared with HIV‐TB patients *p* < 0.05.

### 3.3 T Lymphocytes Mitochondrial Function and Counts Correlated With Inflammatory Factors

**FIGURE 2:**
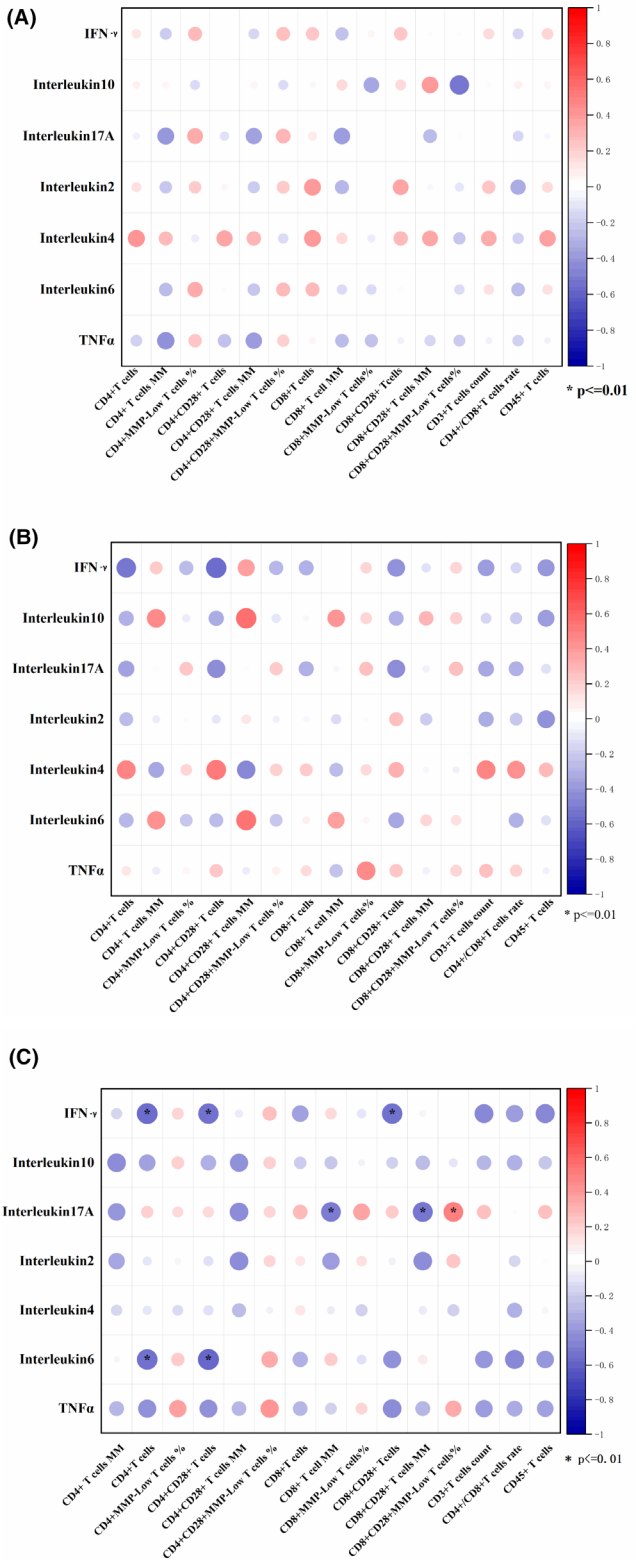
(A) Spearman correlation coefficient visualisation between T lymphocytes mitochondrial function and plasma cytokines in comparison of HIV patients. T lymphocytes' mitochondrial function belonging to various categories were plotted in rows, and cytokines in columns. The significant level was *p* < 0.01; (B) Spearman correlation coefficient visualisation in comparison of HIV‐NTM patients; (C) Spearman correlation coefficient visualisation in comparison of HIV‐TB patients.

We analysed the correlation between the plasma inflammatory factors and T lymphocyte mitochondrial function and counts between different groups (Figure 2). Correlations with a *p*‐value < 0.01 were deemed significant. The correlation heatmap of T lymphocyte mitochondrial function and inflammatory factors is shown in Figure 2A–C. Inflammatory factors, including IFN‐γ, Interleukin‐17A, and Interleukin‐6, were negatively correlated with CD3 + CD4 + T cell count, CD3 + CD4 + CD28+ T cell count, MM of CD3 + CD8+ T cell, CD3 + CD8 + CD28+ T cell count and MM of CD3 + CD8+ T cell but positively correlated with MMP‐low% of CD3 + CD8 + CD28+ T cells in HIV‐TB patients. However, there were no significant differences in other groups.

### 3.4 Alterations in Peripheral Blood T Lymphocytes Function and Inflammatory Factors in HIV Patients With Mycobacterial Infection Occurrence of IRIS

There were no statistically significant differences in CD3 + CD4 + T cell count, MM of CD3 + CD4+ T cell, CD3 + CD4 + CD28+ T cell count, MM of CD3 + CD4 + CD28+ T cells, CD3 + CD8 + T cell count, MM of CD3 + CD8+ T cell, CD3 + CD8 + CD28+ T cell count, MMP‐Low% of CD3 + CD4+ T cells, MMP‐Low% of CD3 + CD8+ T cell, MM of CD3 + CD8 + CD28+ T cell, and MMP‐Low% of CD3 + CD8 + CD28+ T cell in HIV patients with Mycobacterial infection occurrence of IRIS or not (*p* > 0.05). Only MMP‐Low% of CD3 + CD4 + CD28+ T cell and Interleukin 2 were statistically significant (*p* < 0.05), as shown in Table 3.

**TABLE 3:** Comparison of IRIS peripheral blood T lymphocyte mitochondrial function inflammatory factors in patients with HIV and mycobacterial infection.

Groups	IRIS group (*N* = 11)	Non‐IRIS group (*N* = 39)	*p*
CD3 + CD4 + T cell count (pcs/μL)	37.96 (18.92, 110.35)	70.15 (25.35, 217.52)	0.256
MM of CD3 + CD4+ T cell	3.44 (2.42, 4.89)	2.45 (1.67, 3.50)	0.134
MMP‐Low% of CD3 + CD4+ T cell	18.52% (14.53%, 38.75%)	29.34% (19.42%, 45.9%)	0.085
CD3 + CD4 + CD28+ T cell count (pcs/μL)	31.36 (17.52, 103.91)	63.20 (17.53, 165.36)	0.331
MM of CD3 + CD4 + CD28+ T cell	3.11 (2.43, 4.90)	2.62 (1.70, 3.91)	0.178
MMP‐Low% of CD3 + CD4 + CD28 + MMP‐Low T cell	19.05% (14.68%, 38.06%)	29.64% (19.45%, 46.27%)	**0.044**
CD3 + CD8 + T cell count (pcs/μL)	271.75 (89.42, 340.21)	226.74 (140.51, 452.21)	0.682
MM of CD3 + CD8+ T cell	2.75 (1.99, 4.36)	2.40 (1.43, 3.29)	0.174
MMP‐Low% of CD3 + CD8+ T cell	29.7% (18.85%, 38.68%)	37.84% (23.40%, 47.31%)	0.287
CD3 + CD8 + CD28+ T cell count (pcs/μL)	78.08 (31.64, 95.38)	83.18 (40.52, 135.04)	0.331
MM of CD3 + CD8 + CD28+ T cell	2.72 (1.68, 4.45)	2.09 (1.20, 2.83)	0.096
MMP‐Low% of CD3 + CD8 + CD28+ T cell	29.63% (22.09%, 44.68%)	44.59% (33.27%, 53.31%)	0.077
IFN‐γ	3.19 (0.71, 3.85)	1.07 (0.83, 2.28)	0.504
Interleukin10	9.85 (2.71, 18.37)	3.67 (2.44, 10.06)	0.935
Interleukin17A	1.10 (1.01, 1.30)	1.25 (1.02, 2.33)	0.094
Interleukin2	0.41 (0.2, 0.55)	0.54 (0.40, 0.79)	**0.006**
Interleukin4	0.88 (0.74, 1.36)	1.17 (0.91, 1.56)	0.281
Interleukin6	23.61 (9.68, 96.31)	7.98 (5.22, 16.75)	0.210
TNFα	2.48 (1.74, 10.84)	2.73 (1.86, 4.52)	0.309

### 3.5 T Lymphocytes Mitochondrial Function and Inflammatory Factors Predicting IRIS in HIV Patients With Mycobacterial Infection

**FIGURE 3:**
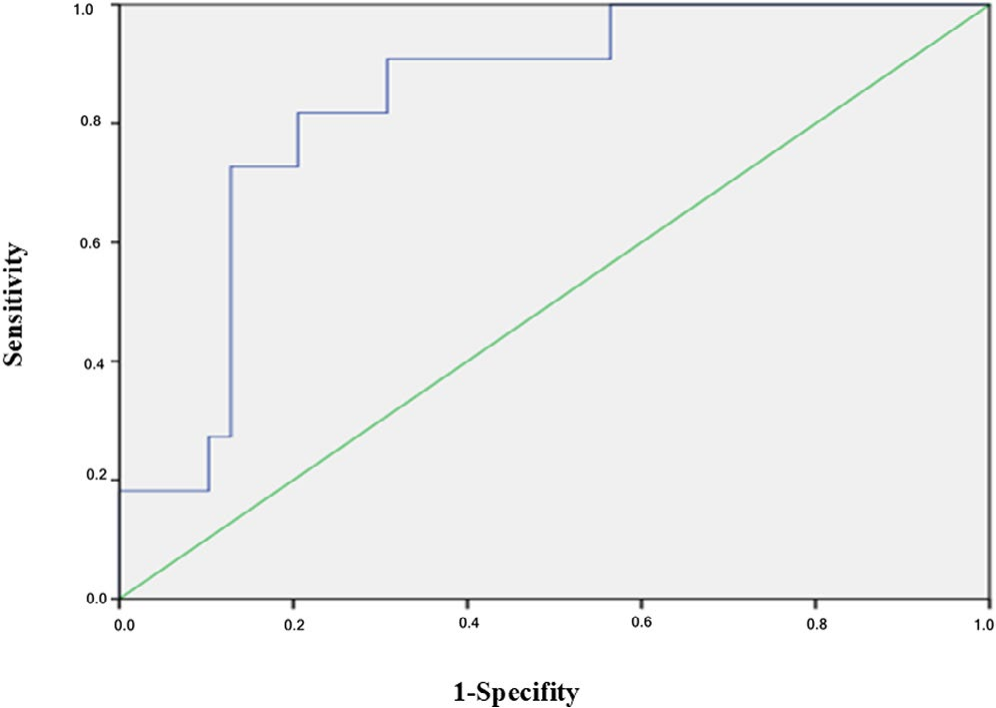
Receiver operating characteristic (ROC) curve of T cells CD3 + CD4 + CD28 + MMP‐Low % and Interleukin‐2 predicting IRIS in HIV patients with Mycobacterial infection. AUC = 0.834 *p* = 0.001.

As shown in Figure 3, 11 HIV patients with mycobacterial infection were defined as IRIS. These patients were on antiviral therapy. Compared to the group without IRIS, the combined MMP‐Low% of CD3 + CD4 + CD28+ T cells and Interleukin‐2 could predict the occurrence of IRIS in HIV patients with Mycobacterial infection, with an AUC of 0.834 (*p* = 0.001).

### 3.6 Changes in Peripheral Blood T Lymphocyte Subsets and Mitochondrial Function in Patients With HIV Complicated With Mycobacterial Infection Before and After Treatment

There were no statistically significant differences in CD3 + CD4 + T cell count, MM of CD3 + CD4+ T cell, CD3 + CD4 + CD28+ T cell count, MM of CD3 + CD4 + CD28+ T cell, CD3 + CD8 + T cell count, MM of CD3 + CD8+ T cell, CD3 + CD8 + CD28+ T cell count, MMP‐Low% of CD3 + CD4+ T cell, MMP‐Low% of CD3 + CD4 + CD28+ T cell, MMP‐Low% of CD3 + CD8+ T cell, MM of CD3 + CD8 + CD28+ T cell, MMP‐Low% of CD3 + CD8 + CD28+ T cell in patients with HIV and mycobacterial infection before and after treatment (*p* > 0.05), as shown in Table 4.

**TABLE 4:** Comparison of peripheral blood T lymphocyte subsets and mitochondrial function in patients with HIV and mycobacterial infection before and after treatment.

Groups	Before treatment (*n* = 10)	After treatment (*n* = 10)	*p*
CD3 + CD4 + T cell count (pcs/μL)	57.83 (24.70, 109.13)	89.11 (51.12, 219.69)	0.285
MM of CD3 + CD4+ T cell	2.21 (1.70, 2.99)	4.02 (1.88, 5.49)	0.139
MMP‐Low% of CD3 + CD4+ T cell	28.8% (17.08%, 47.18%)	24.98% (18.46%, 36.16%)	0.878
CD3 + CD4 + CD28+ T cell count (pcs/μL)	48.35 (21.81, 104.17)	83.08 (26.17, 179.12)	0.445
MM of CD3 + CD4 + CD28+ T cell	2.22 (1.72, 3.00)	4.04 (1.89, 5.63)	0.139
MMP‐Low% of CD3 + CD4 + CD28 + MMP‐Low T cell	28.93% (16.95%, 46.69%)	24.68% (19.55%, 36.05%)	0.959
CD3 + CD8 + T cell count (pcs/μL)	184.22 (100.29, 291.12)	242.23 (88.97, 360.93)	0.333
MM of CD3 + CD8+ T cell	2.35 (1.58, 2.56)	3.01 (1.18, 4.40)	0.169
MMP‐Low% of CD3 + CD8+ T cell	32.17% (22.80%, 39.32%)	32.96% (22.26%, 40.05%)	0.575
CD3 + CD8 + CD28+ T cell count (pcs/μL)	64.69 (18.88, 102.48)	77.16 (29.39, 108.29)	0.333
MM of CD3 + CD8 + CD28+ T cell	1.83 (1.56, 2.78)	2.92 (1.15, 4.98)	0.169
MMP‐Low% of CD3 + CD8 + CD28+ T cell	37.61% (21.16%, 50.81%)	40.25% (25.44%, 52.85%)	0.445

## 4 Discussion

Opportunistic infections are a common cause of death in people living with HIV (PLHIV) [8]. Tuberculosis, a common opportunistic infection in HIV‐infected patients, also has a high mortality rate [9]. In recent years, there has been an increase in the rate of NTM isolation in southwest China, mainly among older patients with high rates of HIV co‐infection [10]. Despite effective combination ART, many patients develop advanced HIV infection (AHD). Patients initiating ART in these clinical conditions may experience paradoxical exacerbations due to an excessive immune response to active infectious agents, a clinical condition known as IRIS, which, if not recognised and treated promptly, can lead to severe morbidity and even mortality [11]. Our study focused on the immune status of the HIV and mycobacterial population, including the development of IRIS.

Mitochondria are energy organelles that are essential for T‐cell homeostasis. MM and MMP‐low are the most recent indicators of mitochondrial function. MM measures the number of protein complexes in the inner mitochondrial membrane respiratory chain, with higher levels indicating enhanced ATP‐producing capacity and stronger dynamics of mitochondrial fusion and division. MMP measures the voltage difference across the mitochondrial membrane, and the higher the MMP level, the lower the MMP‐low, indicating enhanced ATP synthesis, which reflects the cell's metabolic activity [12, 13]. Previously, mitochondrial function has also been studied in HIV‐infected patients, which concluded that the relationship between MM and patients with incomplete immune reconstitution (IIR) was U‐shaped, with a threshold and saturation effect of MM/SD < 2.8, which was negatively correlated with the occurrence of IIR in PLWH after 4, 5, and 6 years of cART treatment. MM observed in the MM/SD ≥ 2.8 group was positively correlated with IIR, possibly due to the overactivation of mitochondria, which play a role in maintaining low‐grade inflammation during latent HIV infection [14]. In our study, comparing HIV‐infected patients (absolute CD4+ T cell count > 200 cells/μL) with patients with mycobacterial infection, it was obvious that compared with HIV‐infected patients, patients with HIV and mycobacterial infection not only had a lower number of T cells but also had lower mitochondrial quality and a higher percentage of cells with lower MMP compared to patients with HIV infection; maybe they were in an immunosuppressed state. In addition, we separated TB and NTM subgroups into mycobacterial infections. We found that compared with HIV‐TB patients, HIV‐NTM patients had significantly lower MM and a higher percentage of cells with lower mitochondrial membrane potential; maybe HIV‐NTM patients were in a more severe immunosuppressed state. This also illustrates the poorer immune status of this group of people with HIV and NTM, which may be the reason for the long‐term latent infection and occurrence of NTM. NTM is a typical opportunistic pathogen in the setting of acquired immunodeficiency syndrome (AIDS). It is more likely to cause disseminated or severe pulmonary disease in hosts with severe immunocompromise [15]. Therefore, the diagnosis of clinically significant NTM disease itself strongly suggests that the underlying immunosuppressive state is already profound, potentially reaching advanced AIDS levels (typically characterised by very low CD4+ T cell counts). The more severe MM loss and the increased proportion of cells with low membrane potential observed in our study directly reflect this deeper state of immune exhaustion characterised by severely impaired T cell function at the level of cellular energy metabolism. Although TB is also severe in people with HIV (PWH), *M. tuberculosis* (MTB) exhibits higher pathogenicity [16] and can cause active disease in PWH with relatively higher CD4+ T cell counts (albeit still indicative of immunosuppression). Consequently, the baseline level of immunosuppression (at diagnosis) in the HIV‐TB cohort may be less extreme overall than in the HIV‐NTM cohort, which is reflected in the relatively milder mitochondrial impairment observed. Furthermore, NTM infection (especially *Mycobacterium avium* complex–MAC) often follows a more chronic course in PWH and may persist for a longer duration before diagnosis or the onset of severe symptoms. This persistent antigen exposure can drive more profound T cell exhaustion [17]. As a result, HIV‐NTM patients may experience a longer period of cumulative immune system ‘wear and tear’, leading to more severe accumulation of mitochondrial damage. While TB infection can also become chronic, its higher pathogenicity frequently prompts earlier symptom manifestation and medical intervention. This potentially shorter pre‐treatment disease duration may mean that immune exhaustion, particularly at the mitochondrial level, does not accumulate to the same depth as observed in long‐standing NTM infections.

We also found that inflammatory factors, including IFN‐γ, Interleukin‐17A and Interleukin‐6, were negatively correlated with CD3 + CD4 + T cell count, CD3 + CD4 + CD28+ T cell count, MM of CD3 + CD8+ T cell, CD3 + CD8 + CD28+ T cell count and MM of CD3 + CD8+ T cell but positively with MMP‐low% of CD3 + CD8 + CD28+ T cells in HIV‐TB patients. However, there were no significant differences in other groups. The core of our explanation hinges on the profoundly distinct immunopathological environment created by the co‐occurrence of active TB and HIV infection. This environment is characterised by: (1) Extreme and Chronic Immune Activation/Inflammation [18]: HIV‐TB patients experience relentless, high‐grade inflammation driven synergistically by HIV viral replication, *M. tuberculosis* infection and their interplay. This results in persistently elevated levels of pro‐inflammatory cytokines, including IFN‐γ, IL‐17A and IL‐6, far exceeding levels typically seen in HIV mono‐infection (HIV group). (2) Profound T Cell Exhaustion [19]: Chronic antigenic stimulation from both pathogens drives severe T cell exhaustion. (3) Metabolic Dysregulation and Mitochondrial Dysfunction [20]: chronic inflammation and immune activation are intrinsically linked to cellular metabolic reprogramming and mitochondrial dysfunction. T cell effector functions critically depend on mitochondrial ATP production and biosynthetic intermediates. A low mitochondrial membrane potential (MMP) is a key indicator of impaired mitochondrial function, cellular dysfunction and increased susceptibility to apoptosis. (4) Impaired Immune Reconstitution: Even under antiretroviral therapy (ART), immune recovery in HIV‐TB patients is often suboptimal compared to HIV mono‐infected individuals [21]. Inverse Correlations (Cytokines vs. T Cell Counts): High levels of pro‐inflammatory cytokines (IFN‐γ, IL‐17A, IL‐6) are markers of persistent, intense immune activation and pathological inflammation. In this extreme environment, these cytokines themselves or the downstream effects they induce (such as triggering activation‐induced cell death (AICD), promoting exhaustion, suppressing thymic output or disrupting lymphocyte homing) may directly or indirectly lead to the reduction or impaired maintenance of specific T cell subsets (e.g., CD4+, CD8 + CD28+) [18]. Therefore, the observed inverse correlations likely reflect the depleting or suppressive effect of the pathological inflammatory milieu on the T cell pool, rather than a positive stimulatory role of these cytokines on these cells within this specific context. This phenomenon shares similarities with ‘inflammation‐induced lymphopenia’ observed in human endotoxemia [22] and sepsis [23]. Inverse Correlations (Cytokines vs. T Cell Mitochondrial Mass (MM)): MM reflects mitochondrial content/biogenesis. The inverse correlation may indicate that chronic inflammatory stress suppresses the metabolic adaptability of T cells or promotes mitophagy/clearance, leading to poor maintenance of the mitochondrial network. This is consistent with reports of T cell metabolic dysfunction in pulmonary tuberculosis and chronic inflammatory states [24]. Crucial Positive Correlation (Cytokines vs. Percentage of CD8 + CD28+ T cells with Low MMP): This is the most distinctive finding of our study and provides strong support for the pathological environment explanation. Low MMP is a direct indicator of severely impaired mitochondrial function and a pre‐dysfunctional/apoptotic cellular state. The positive correlation between inflammatory cytokines (e.g., IFN‐γ) and the percentage of CD8 + CD28+ T cells exhibiting low MMP strongly suggests that, within the extreme inflammatory context of HIV‐TB co‐infection, high levels of pro‐inflammatory signals are not promoting the functional activation of CD8 + CD28+ T cells but are instead closely associated with the dysfunction and reduced survival capacity of these crucial effector cells. This aligns with studies showing that exhausted T cells often exhibit mitochondrial dysfunction [25]. In other words, the highly inflammatory environment exerts a ‘toxic effect’ on effector T cells that should be functional, leading to their functional collapse (manifested by decreased MMP), and the extent of this collapse correlates positively with the inflammatory burden.

In addition, after dividing the patients into the IRIS group and the non‐IRIS group, we found that the percentage of CD3 + CD4 + CD28+ MMP‐Low T cells in the patients with IRIS was lower than that in the non‐IRIS group, which means that their immune status was relatively active. That said, the occurrence of IRIS is not necessarily a bad outcome. Finally, there was no significant difference in the number of T lymphocyte subsets and mitochondrial function in the peripheral blood of patients with Mycobacterium HIV infection before and after paired treatment. It can be seen that antimycobacterial therapy within six months could not significantly affect the number of T lymphocyte subsets and mitochondrial function in peripheral blood.

Due to the limited number of cases and clinical phenomena, our study inevitably has some limitations. First, more precise clinical thresholds for MM and MMP‐low in HIV‐infected patients require large‐scale studies. Second, the study is a single‐centre prospective study. This study is an exploratory pilot study to investigate the role of T lymphocyte mitochondrial function and peripheral blood inflammatory factors in patients co‐infected with HIV and mycobacteria. Limited research resources (time, funding, accessibility to the specific population); recruiting large samples is very challenging; consequently, a small sample size was employed. However, the study adopted a prospective design, implemented strict data collection and quality control processes, utilised gold‐standard diagnostic methods to confirm disease status and conducted detailed subgroup analyses to ensure the internal validity of the results. A large‐scale multicentre study with expanded enrolment will provide more data needed for such studies. Finally, for future studies, we suggest a longer longitudinal study to track changes in peripheral blood T lymphocyte subsets and mitochondrial function in patients with HIV mycobacterial infection with prolonged antimycobacterial therapy.

In summary, the immune status of patients co‐infected with HIV and mycobacteria is significantly inferior to that of patients with HIV infection alone, not only in terms of cell number but also in mitochondrial status. This immune state may be associated with the occurrence of inflammation. Therefore, the mechanism of immune inflammation in these patients still needs to be further explored to help control the occurrence and treatment of the disease.

## Author Contributions

**Mengyan Wang:** investigation (equal), methodology (equal), writing – original draft (equal), writing – review and editing (equal). **Xiaotian Dong:** investigation (equal), methodology (equal). **Hu Wan:** investigation (equal), methodology (equal). **Jinchuan Shi:** investigation (equal), methodology (equal). **Lu Hui:** investigation (equal), methodology (equal). **Shourong Liu:** conceptualization (equal), supervision (equal), writing – review and editing (equal). **Jun Yan:** conceptualization (equal), supervision (equal), writing – review and editing (equal).

## Ethics Statement

This study was approved by the Ethics Committee of Xixi Hospital of Hangzhou in China (Approval no:2022059).

## Consent

All patients provided written informed consent. All procedures and methods are carried out in accordance with relevant international guidelines and regulations to reduce the physical discomfort of the subjects.

## Conflicts of Interest

The authors declare no conflicts of interest.

## Supporting information


**Data S1:** jcmm70832‐sup‐0001‐SupplementaryAppendix.docx.

## Data Availability

Data will be made available on request.
