# Supplementary material for: Altered T Lymphocytes Mitochondrial Function and Inflammatory Factors of Peripheral Blood in HIV Patients With Mycobacterial Infection
Source: J Cell Mol Med. 2025 Sep 11;29(17):e70832. doi: 10.1111/jcmm.70832 (PMC12425818; doi:10.1111/jcmm.70832)
Supplement: Supplementary file 1 — Data S1: jcmm70832‐sup‐0001‐SupplementaryAppendix.docx. [file JCMM-29-e70832-s001.docx]

## Graphical analysis

| 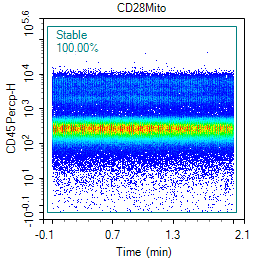 | Select the data stabilization region |
| --- | --- |
| 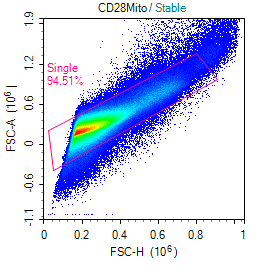 | The adhesion signal was removed using FSC-H/FSC-A plots |
| 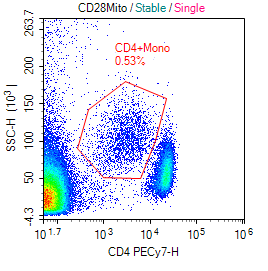 | Circle CD4 monocytes |
| 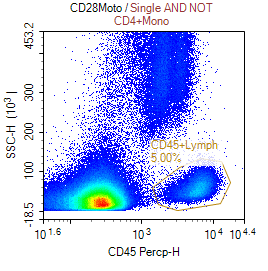 | After monocytes are excluded, CD45 lymphocytes are circled |
| 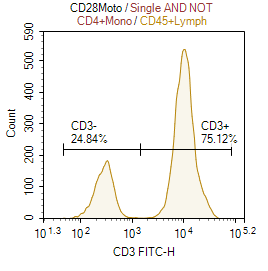 | The two-region gate divided the CD3 negative and positive cell populations |
| 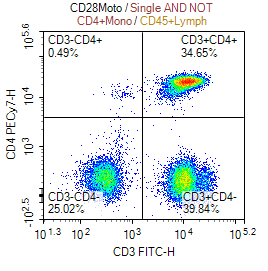 | Quadrant gates divide CD3 CD4 cells |
| 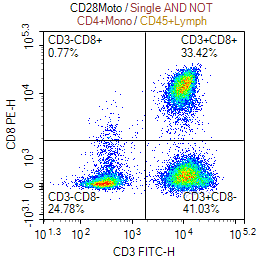 | Quadrant gates divide CD3 CD8 cells |
| 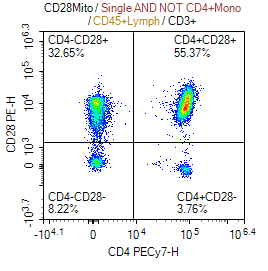 | CD3 clusters were read, and the quadrant gate was divided into CD4 CD28 cells |
| 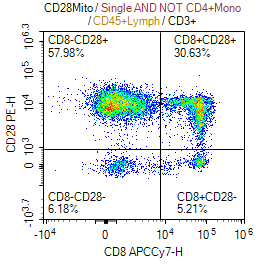 | CD3 grouping was read, and the quadrant gate partitioned CD8 CD28 cells |
| 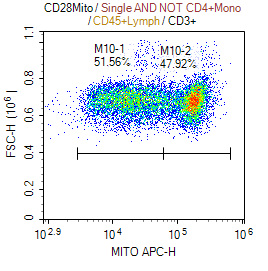 | CD3 partitioning was read, and the two-region gate partitioned Mito negative and positive partition |
| 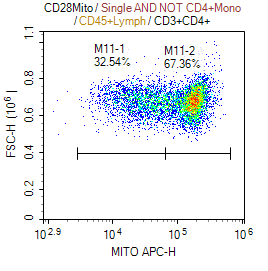 | CD3 CD4 partitions were read, and the biregional gate partitioned Mito negative and positive partitions |
| 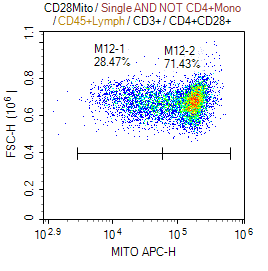 | CD4 CD28 grouping was read, and the biregional gate divided the Mito negative and positive partition |
| 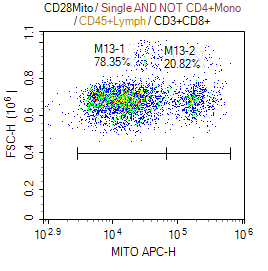 | CD3 CD8 partitions were read, and the biregional gate partitioned Mito negative and positive |
| 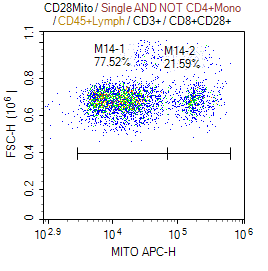 | CD8 CD28 grouping was read, and the biregional gate partition was divided into Mito negative and positive partitions |
| 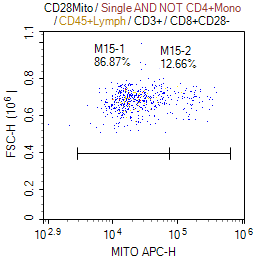 | CD8 CD28- grouping was read, and the biregional gate divided the Mito negative and positive partition |
|  | Check whether the compensation between the fluorescence channels is appropriate |
